# Functionalized nano SiO_2_ reinforced gelatin–PVA hydrogels for sustainable wood adhesion

**DOI:** 10.1039/d6lf00152a

**Published:** 2026-05-28

**Authors:** Sogand Abbaspoor-Zanjani, Carlo Di Bernardo, Jenny Flores Garcia, Mengjiao Wang, Massimo Messori, Camilla Noè, Teresa Gatti

**Affiliations:** a Department of Applied Science and Technology, Politecnico di Torino Corso Duca degli Abruzzi 24 10129 Torino Italy sogand.abbaspoor@polito.it camilla.noe@polito.it teresa.gatti@polito.it

## Abstract

The development of sustainable wood adhesives that avoid formaldehyde-based chemistries remains an important challenge for the wood composite industry. In this work, bio-based adhesive hydrogels based on gelatin and poly(vinyl alcohol) (PVA) were reinforced with surface-functionalized silica nanoparticles to enhance their rheological and adhesive performance. Silica nanoparticles were synthesized *via* a modified Stöber sol–gel process and functionalized with 3-aminopropyltriethoxysilane (APTES) to introduce surface amine groups capable of interacting with the polymer matrix. The nanoparticles were incorporated into gelatin/PVA blends with different polymer ratios (2 : 1–5 : 1 gelatin : PVA), and the resulting hybrid systems were characterized using SEM, DLS, FTIR, TGA, and rheological analysis. The incorporation of SiO_2_-APTES significantly reinforced the hydrogel network, increasing storage modulus and viscosity while maintaining pronounced shear-thinning behaviour suitable for adhesive applications. The strongest improvement was observed for the 5 : 1 gelatin : PVA formulation, where nanoparticle incorporation increased the storage modulus from ∼240 to ∼450 Pa. Lap-shear testing on wood substrates revealed a maximum adhesion strength of 735 ± 25 kPa, corresponding to a 71% increase compared with the unfilled gelatin/PVA adhesive and a 122% increase relative to gelatin alone. These improvements are attributed to a combination of proposed interactions, including hydrogen bonding, ionic interactions, and possible mechanical interlocking between the functionalized nanoparticles, polymer network, and wood surface. The results demonstrate that silica-reinforced gelatin/PVA hydrogels represent a promising formaldehyde-free, bio-based adhesive platform for sustainable wood bonding applications.

## Abstract 2

A gelatin/PVA/SiO_2_ hydrogel adhesive is developed for wood bonding. Enhanced interfacial bonding between the adhesive matrix and hydroxyl-rich wood surfaces leads to improved adhesion performance.

## Graphical abstract

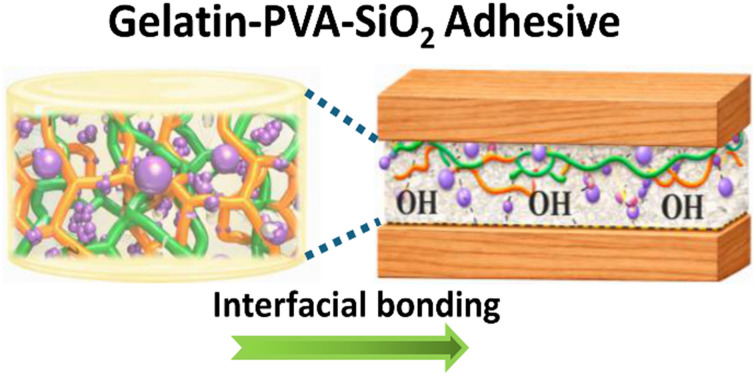

## Introduction

Wood adhesive technology is undergoing a rapid transition from petroleum-derived synthetic resins toward eco-friendly, bio-based formulations. This shift is driven by increasingly strict regulations targeting formaldehyde emissions and volatile organic compounds (VOCs) in wood adhesive systems.^1^ Recent advances in bio-based wood adhesives include mussel-inspired gelatin/dopamine hydrogels^2^ and hydrogel tapes,^3^ tannin/epoxy systems,^4^ modified lignin-based adhesives,^5^ and soy protein–catechol hybrids,^6^ all designed to improve bonding strength, water resistance, and sustainability in wood-based materials. Although natural adhesives have demonstrated performance approaching that of synthetic counterparts in several cases, their adhesive properties still require further improvement.

Blending biopolymers with synthetic components has emerged as an effective strategy to generate synergistic effects that enhance adhesiveness and mechanical strength while maintaining sustainability. Hybrid protein-synthetic systems exemplify this approach. Morsi *et al.*^7^ incorporated gelatin into poly(vinyl acetate) (PVAc) latex adhesives and reported improved adhesion strength and reduced water permeability relative to neat PVAc. These improvements were attributed to enhanced intermolecular interactions between gelatin polypeptide chains and the PVAc matrix, resulting in a denser adhesive film. Gelatin, a protein-based polymer, exhibits strong intrinsic adhesion but suffers from brittleness and high hydrophilicity. When blended with poly(vinyl alcohol) (PVA), a flexible polymer with excellent film-forming capability, the resulting composites display significantly improved adhesiveness and mechanical strength.^8^ Gelatin/PVA hydrogels prepared *via* freeze–thaw cycles or citric acid crosslinking demonstrate enhanced tensile strength and stable adhesive performance.^9,10^ Furthermore, adding PVA to gelatin-rich systems slows degradation and enhances mechanical integrity, with optimal ratios yielding durable, moisture-resistant adhesive films.^11,12^ These enhancements arise primarily from extensive intermolecular hydrogen bonding between the hydroxyl groups of PVA and the amino/carboxyl groups of gelatin.^10^ Consequently, gelatin/PVA blends have attracted interest for applications including wood bonding, paper lamination, and biomedical materials.

Several studies have demonstrated the versatility of gelatin/PVA systems across application domains. Nguyen *et al.*^13^ optimized gelatin/PVA crosslinking using genipin to produce hydrogels with compressive strength of around 7.5 MPa and significantly reduced swelling. Wattanavijitkul *et al.*^14^ reported that gelatin/PVA bio-coatings crosslinked with glutaraldehyde formed durable adhesive interfaces. Fernandes *et al.*^15^ developed biodegradable gelatin/PVA films crosslinked by glutaraldehyde or UV irradiation for packaging applications and observed significant improvements in tensile strength and flexibility due to enhanced hydrogen bonding. Overall, these findings demonstrate that gelatin/PVA systems can be tailored through formulation and crosslinking to achieve desirable mechanical strength and moisture stability.

Despite these advances, a significant knowledge gap remains regarding the optimization of these blends for wood adhesion, a demanding application requiring high shear strength and specific curing behaviour.^16^ To address the intrinsic limitations of bio-based matrices, incorporation of nanofillers into polymer adhesives has emerged as an effective reinforcement strategy. Kaboorani and Riedl^17^ investigated the incorporation of montmorillonite nanoclay into poly(vinyl acetate) (PVAc) wood adhesives and observed improvements in thermal stability as well as dry and wet shear strength. Chaabouni and Boufi^18^ incorporated cellulose nanofibrils (CNF) into PVAc adhesives and observed substantial increases in shear strength at increasing CNF loadings, particularly near 10 wt% loading, though higher concentrations increased viscosity. Similarly, Wang *et al.*^19^ incorporated nano-SiO_2_ into starch-based wood adhesives and achieved approximately 50% increase in dry bonding and 84% improvement in wet bonding strength. These studies demonstrate that nanoscale fillers can significantly enhance mechanical reinforcement and moisture resistance in bio-based adhesive systems.

Beyond filler incorporation, nanoparticle surface modification plays a critical role in optimizing interfacial interactions. Moghaddam and Tutunchi^20^ demonstrated that silane-functionalized SiO_2_ nanoparticles improve nanoparticle dispersion and strengthen organic–inorganic interfacial interactions within polymer matrices within a UV-curable polyurethane acrylate adhesive system. Although not specific to wood adhesives, the study provides mechanistic insight into how surface-functionalized SiO_2_ can enhance composite adhesive performance.

While SiO_2_ nanoparticles have shown effectiveness in biomedical^21,22^ and structural adhesive systems,^23^ their systematic integration into gelatin/PVA matrices for wood bonding applications remains limited, particularly when surface functionalization is considered. Therefore, a significant knowledge gap exists in the development of gelatin/PVA adhesives reinforced with functionalized SiO_2_ NPs for high-performance wood adhesion.

**Scheme 1:**
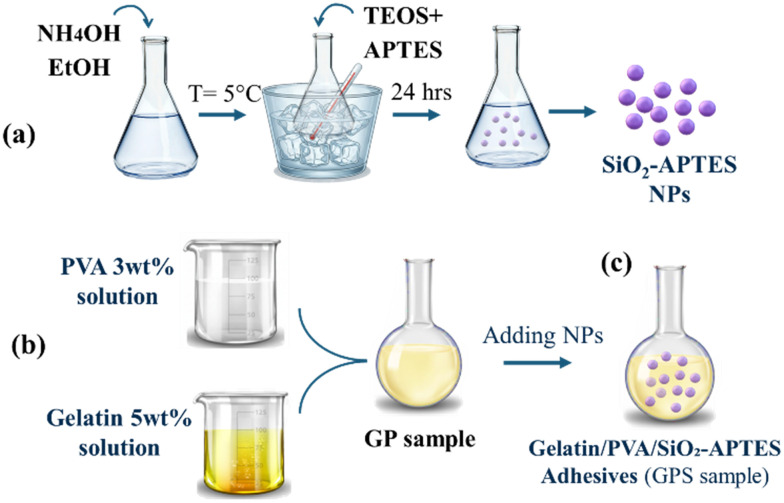
Experimental procedure for the preparation of SiO_2_-reinforced gelatin/PVA-based adhesives: (a) synthesis of SiO_2_-APTES NPs; (b) preparation of the gelatin/PVA blend; and (c) incorporation of SiO_2_-APTES NPs into the gelatin/PVA adhesives.

In this study, APTES-functionalized SiO_2_ (SiO_2_-APTES) NPs were synthesized and incorporated into gelatin/PVA adhesives with varying gelatin ratios (Scheme 1). The viscoelastic and shear-thinning behaviors of the resulting hydrogels were evaluated through rheological analysis, followed by lap shear testing on wood substrates. This work demonstrates that SiO_2_ functionalization promotes enhanced molecular interactions and possibly mechanical interlocking at the adhesive–wood interface. The proposed strategy provides a robust, durable, and formaldehyde-free bio-based adhesive system, offering a sustainable pathway for next-generation wood composite technologies.

## Experimental

### Materials

Commercial Gelatin and poly(vinyl alcohol) (MW: 30 000–70 000), ethanol (EtOH, 98%), aqueous ammonia (NH_4_OH, 28–30%), tetraethyl orthosilicate (TEOS, 98%), 3-aminopropyltriethoxysilane (APTES, 99%, Sigma-Aldrich) were purchased from Sigma-Aldrich and used without further purification. Water used was Milli-Q grade.

### Synthesis of SiO_2_-APTES nanoparticles (SiO_2_-APTES NPs)

SiO_2_-APTES NPs were synthesized using a sol–gel process adapted from the Stöber method.^24^Scheme 1(a) schematically illustrates the synthesis route for SiO_2_-APTES NPs. In this process, a mixture of ammonium hydroxide solution and ethanol was stirred for 20 minutes. TEOS, as a silica precursor and APTES were pre-mixed in a 1 : 1 volume ratio and then added dropwise to the solution, which was maintained at 5 °C using an ice bath to control nanoparticle growth. The temperature was monitored using a laboratory thermometer and adjusted by controlled addition of ice, while continuous stirring ensured a homogeneous temperature distribution throughout the solution. The use of reduced temperature was intentional, as it slows down the hydrolysis and condensation kinetics of TEOS, enabling improved control over nucleation, particle growth, and surface functionalization. All syntheses were conducted under identical experimental conditions to ensure reproducibility. The reaction proceeded under continuous stirring for 2 hours at this controlled temperature. Subsequently, the ice bath was removed, and stirring continued for an additional 24 hours to facilitate nanoparticle formation. The resulting nanoparticles were collected *via* centrifugation, thoroughly washed with ethanol to remove residual ammonia and minimize water content, and finally dried under vacuum.

Preparation of gelatin/PVA/SiO_2_-APTES hybrid adhesive system adhesive formulations. Scheme 1b presents the fabrication process of the gelatin/PVA/SiO_2_-APTES hybrid adhesive system. To effectively mix PVA and gelatin, first PVA 3 wt% solution was prepared by dissolving PVA in distilled water heated to 60 °C, stirring continuously for 2 hours until fully dissolved. In a separate beaker, gelatin was dissolved in water heated to 70 °C, gradually adding the powder while stirring gently for 2 hours to prevent foam formation to prepare gelatin 5 wt% solution. Once both solutions were prepared, the warm gelatin solution was slowly poured into the PVA solution while maintaining a temperature of 50–60 °C, stirring continuously for 30 minutes to ensure uniform mixing. After mixing, the solution was gradually cooled while stirring to prevent phase separation. To improve dispersion and homogeneity, ultrasonication was applied before storage. Once the polymer blend was fully prepared, SiO_2_-APTES NPs were incorporated into the solution. To prevent agglomeration, the nanoparticles were first dispersed in a 15 ml of distilled water under continuous stirring and ultrasonication. A fixed amount of this dispersion (1 wt% relative to total polymeric solution weight) was then gradually added to the gelatin/PVA mixture while maintaining constant gentle stirring for 4 hours. This prolonged mixing ensured improved dispersion within the polymer matrix of the nanoparticles and promoted effective bonding with the polymeric matrix. Scheme 1(c) shows the final prepared adhesive solutions. The nanoparticle loading was fixed at 1 wt% to isolate the effects of surface functionalization and polymer composition on the adhesive performance. Systematic optimization of nanoparticle loading is beyond the scope of the present study and will be addressed in future work.

The samples are coded to distinguish between base and filled formulations. GP refers to the unfilled gelatin/PVA mixtures, while GPS denotes the corresponding SiO_2_-APTES filled composites. A 2 : 1 gelatin : PVA weight ratio is labeled as GP2, with its nanoparticle-filled counterpart designated as GPS2. This naming convention is consistently applied to all other ratios studied, including 3 : 1 (GP3/GPS3), 4 : 1 (GP4/GPS4), and 5 : 1 (GP5/GPS5) of gelatin : PVA ratio.

### Materials characterization

Dynamic light scattering (DLS) was used to determine hydrodynamic diameter and distribution of the SiO_2_ NPs before and after APTES functionalization using Litesizer DLS (Anton Paar). Dispersions (≈0.10 mg mL^−1^) were prepared in filtered ethanol/water (EtOH : H_2_O, 70 : 30 v/v) and sonicated for 10 min in a bath sonicator to minimize agglomeration. Measurements were performed at 25 °C using a backscatter detection angle of 173°, automatic attenuation, and refractive indices of 1.33 (dispersant) and 1.46 (SiO_2_). Each sample was equilibrated for 120 s prior to analysis and recorded in triplicate (≥10 runs per replicate, 10s per run). Size distributions were evaluated with cumulants analysis (*z*-average) and number-weighted distributions reported after instrument software conversion; results are expressed as mean ± SD. The images of SiO_2_ NPs were acquired by scanning electron microscopy using TESCAN SEM with an acceleration voltage of 5 kV. Dilute nanoparticle suspensions in ethanol were drop-cast onto carbon-coated copper grids and dried under ambient conditions. Fourier-transform infrared (FT-IR) spectra were collected in attenuated total reflectance (ATR) mode using Thermos Scientific Nicolet iS50b FTIR Spectrometer to confirm surface functionalization. Dry powders of bare SiO_2_ and SiO_2_-APTES were pressed onto the diamond ATR crystal. Spectra were recorded from 4000–400 cm^−1^, 32 scans per spectrum at room temperature. A fresh background spectrum of air was acquired before each measurement. Thermogravimetric analysis (TGA) was performed using Netzsch STA409PC instrument to observe the presence of APTES grafting on the surface of SiO_2_ NPs. 5 mg of dry powder was placed in a pan and heated from 25 to 800 °C at 10 °C min^−1^.

The surface area and pore characteristics of SiO_2_ and APTES-functionalized SiO_2_ NPs were determined using nitrogen adsorption–desorption measurements on a Micromeritics ASAP 2020 Plus surface area analyzer. Prior to analysis, the samples were degassed under vacuum at 363 K for 1 h followed by 423 K for 8 h to remove adsorbed moisture and impurities. The adsorption–desorption isotherms were recorded at 77 K. The specific surface area was calculated using the Brunauer–Emmett–Teller (BET) method, while pore characteristics were obtained from Barrett–Joyner–Halenda (BJH) analysis based on the desorption branch of the isotherm.

### Rheological characterization

Gelatin/PVA formulations were tested on a modular compact rheometer using MCR 702e MultiDrive, Anton Paar (Graz, Austria) in parallel-plate configuration (25 mm, 1 mm gap). Samples containing different proportions of gelatin : PVA and SiO_2_-APTES NPs were analyzed in a frequency-sweep experiment at 25 °C, setting the strain at 1% and varying the angular frequency from 0.1 to 100 rad s^−1^. The storage modulus (*G*′) values of the samples were then recorded. Additionally, steady-shear viscosity curves were obtained with a shear rate ranging from 0.1 to 100 1/s. To highlight the effect of SiO_2_-APTES NPs, the difference in storage modulus between nanoparticle-containing samples (GPS) and their corresponding nanoparticle-free controls (GP) was determined using the following equation and plotted *versus* time to illustrate how nanoparticles influence both the magnitude and dynamic behavior of elasticity.Δ*G*′ = *G*′(GPS) − *G*′(GP)

### Lap shear test

Lap-shear strength of the formulated bio-based adhesives was measured using a Zwick-Roell BT1-FR100 universal testing machine. Plywood plates (150 × 40 × 3 mm) were bonded with an overlap length *L* = 50 full width *b* = 40 mm, giving a nominal bonded area of 2000 mm^2^. The bonding surfaces of the adherends were cleaned to remove contaminants. The adhesive was then applied uniformly to one substrate using an applicator, after which the second substrate was positioned, and the assembly was allowed to cure for 5 hours. A calibrated load cell at 10 kN, recorded force continuously. All tests and curing steps were conducted under ambient laboratory conditions with temperatures of 25 ± 1 °C and relative humidity of (50 ± 5)%. To provide further insight into the substrate characteristics, surface topography and 2D roughness map of the plywood were characterized using a confocal optical profiler (S neox, Sensofar, Spain). The substrate exhibited a heterogeneous morphology with an average roughness of *S*_a_ = 2.04 ± 0.28 μm, *S*_q_ = 3.41 ± 0.04 μm, and a peak-to-valley height of *S*_z_ = 141 ± 3.5 μm, reflecting the intrinsic porosity and microstructure of the wood. The 3D roughness map is provided in SI (Fig. S1). A quasi-static loading rate, corresponding to a crosshead speed of 1.2 mm min^−1^ was applied. Each test was repeated three times under identical conditions, and average values were reported with standard deviations. Finally, the lap-shear strength *τ*_LS_ was calculated from the maximum load *F*_max_ divided by the nominal bonded area (*A*_b_):
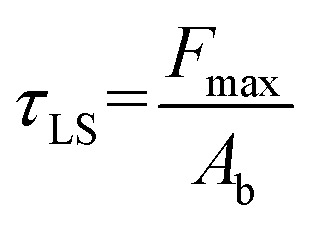


## Results and discussion

The objective of this research is to systematically investigate the influence of SiO_2_ NPs on the adhesive property of gelatin/PVA. Stöber method is used to synthesize SiO_2_ NPs first. To synthesize SiO_2_-APTES NPs appropriate for this task, we used a simple sol–gel method published before to functionalize SiO_2_ with APTES (Scheme 1a). This approach caused successful grafting of SiO_2_ with APTES, which improved higher possibility to combine with gelatin/PVA (Scheme 1(b)).

### SEM and DLS characterization

**Fig. 1:**
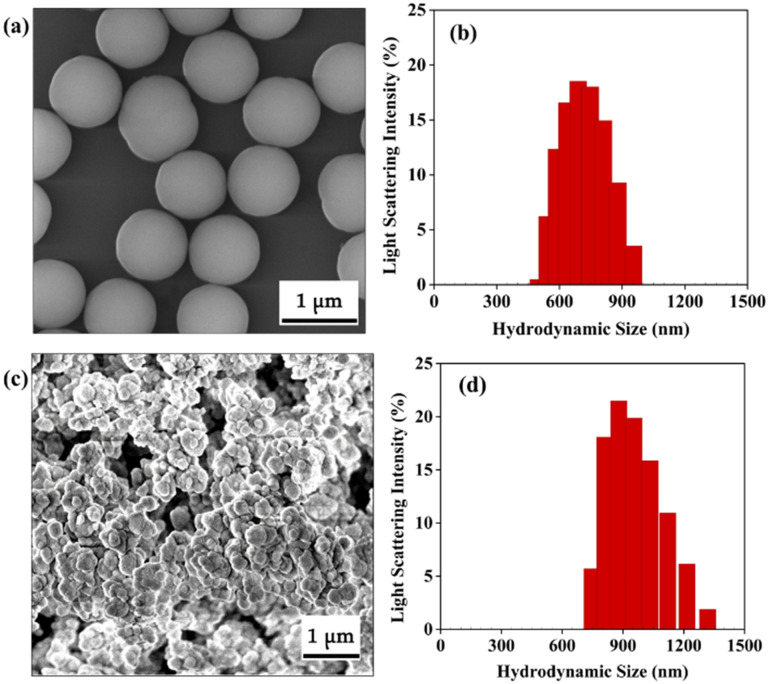
SEM image (a) and size distribution from DLS (b) of SiO_2_ NPs. SEM image (c) and size distribution from DLS (d) of SiO_2_-APTES NPs.

Fig. 1(a) presents the SEM image of the synthesized SiO_2_ NPs, which appear spherical and uniformly distributed. Fig. 1(b) shows the DLS results, indicating a polydisperse sample with a primary peak between 720 and 780 nm, which is in accordance with the size shown in Fig. 1(a). Fig. 1(c) displays the SEM image of the SiO_2_-APTES NPs, revealing a cauliflower-like morphology characterized by clustered particles. Owing to the organic APTES on the surface, the functionalized SiO_2_ is unlikely to show clear round shape. Fig. 1(d) illustrates their size distribution, with a main peak around 900–1000 nm. The increased hydrodynamic size and broader size distribution suggest that APTES modification enlarged the particles by polymer shell.

### FTIR: structural verification of APTES

**Fig. 2:**
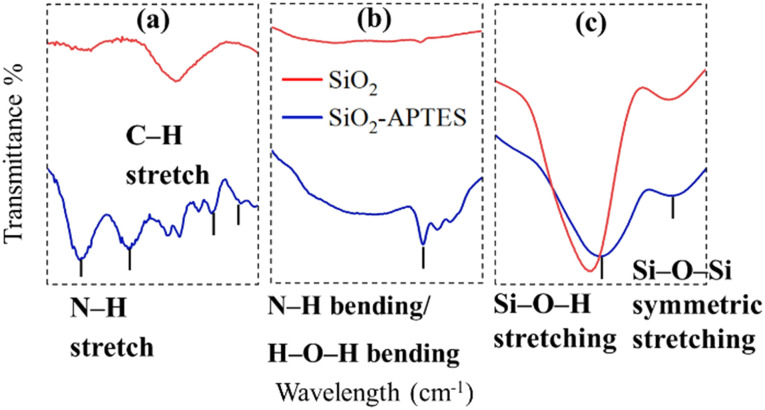
Three main spectral regions in the FT-IR of SiO_2_ and SiO_2_-APTES NPs: (a) O–H/N–H and C–H stretching vibrations (2800–3700 cm^−1^), (b) N–H bending vibrations (1450–1750 cm^−1^), and (c) Si–O–Si framework vibrations (800–1250 cm^−1^).

The full FTIR spectra of SiO_2_ and SiO_2_-APTES NPs are reported in the SI (Fig. S2). Three important regions with the signal of critical functional groups are magnified for better analysis as presented in Fig. 2. In region (a) (2800–3700 cm^−1^) both samples exhibit a broad absorption band centered around 3200–3700 cm^−1^, corresponding to O–H stretching vibrations of adsorbed water.^25^ In the SiO_2_-APTES spectrum, this region shows increased intensity and a slight shift due to overlapping N–H stretching vibrations from the amine (–NH_2_) groups introduced by APTES.^26^ Additionally, weak bands appearing at 2850–2950 cm^−1^ in the SiO_2_-APTES spectrum correspond to C–H stretching vibrations from the propyl chains (−(CH_2_)_3_–NH_2_), confirming the presence of organic functional groups. In region (b) (1450–1750 cm^−1^) a distinct broad peak around 1550–1650 cm^−1^ is observed in the SiO_2_-APTES spectrum, which is attributed to N–H bending vibrations of primary amine groups.^26^ This peak often overlaps with the H–O–H bending vibrations of adsorbed water. The enhanced intensity of this band compared to SiO_2_ supports the successful introduction of amine functionalities on the nanoparticle surface. Region (c) (800–1250 cm^−1^) corresponds to the characteristic Si–O–Si framework vibrations of the SiO_2_ network. Both spectra display a strong asymmetric stretching band at 1000–1250 cm^−1^ and a symmetric stretching band near 800 cm^−1^.^24^ The SiO_2_-APTES spectrum shows a marked decrease of the shoulder near 950–960 cm^−1^, suggesting that surface silanol groups were consumed during the condensation reaction with APTES.^26^ Overall, the spectral variations across regions (a)–(c) provide clear evidence for the successful surface functionalization of SiO_2_ NPs with APTES.

Thermogravimetric analysis of bare SiO_2_ and SiO_2_–APTES NPs was conducted to evaluate their thermal degradation behaviour (Fig. S3). The SiO_2_–APTES sample exhibited an additional weight loss compared to SiO_2_, which is attributed to the decomposition of organic aminopropyl groups and confirms the successful grafting of APTES onto the SiO_2_ surface. Moreover, nitrogen adsorption–desorption measurements were performed to investigate the textural properties of the samples. The bare SiO_2_ NPs showed a BET surface area of 5.01 m^2^ g^−1^, which decreased to 3.61 m^2^ g^−1^ after APTES functionalization, indicating partial coverage of the SiO_2_ surface and reduced accessibility of nitrogen adsorption sites. Detailed adsorption–desorption isotherms and pore size distribution analyses are provided in the SI (Table S2 and Fig. S4).

### Rheological characterization of gelatin/PVA based adhesives

**Fig. 3:**
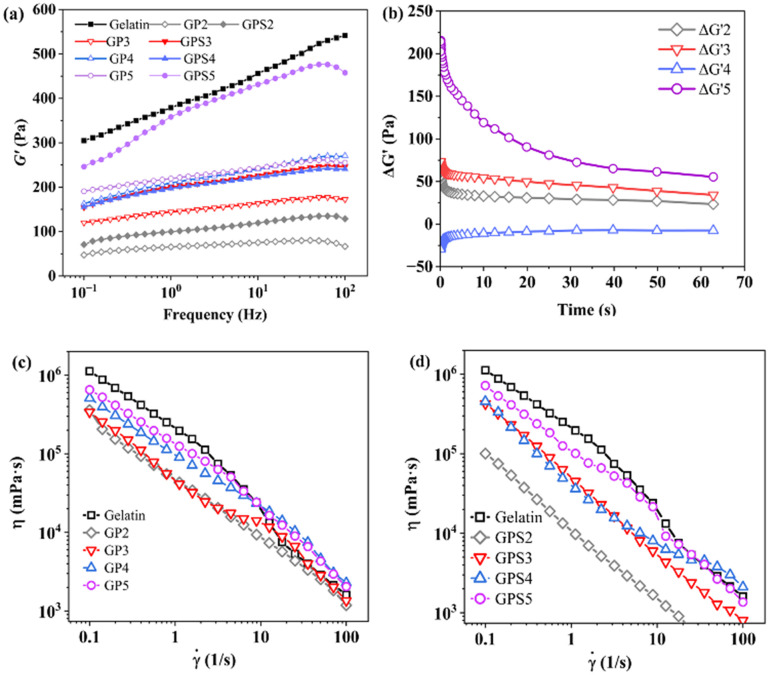
Rheological analysis of gelatin 5 wt%, and gelatin/PVA systems; GPS: with and GP: without SiO_2_-APTES (a) frequency dependence of storage modulus (*G*′) for gelatin and gelatin/PVA samples (b) time evolution of Δ*G*′. (c) Shear viscosity (*η*) as a function of shear rate (*γ̇*) for gelatin, GP samples and (d) for GPS samples.

SiO_2_-APTES NPs were then added to the gelatin/PVA adhesives through simple mixture and sonication process. For comparison, pure gelatin/PVA was fabricated with the same experimental conditions. Tuning the rheology of the adhesives is critical for process-specific applications. In this study, the impact of SiO_2_-APTES NPs on the viscoelastic properties and shear-thinning behavior of the system was investigated. Oscillatory frequency sweeps in Fig. 3(a) illustrate the variation of storage modulus (*G*′) with frequency for all the samples. The rheological measurements clearly show that, compared to each gelatin/PVA sample, the corresponding gelatin/PVA/SiO_2_-APTES system shows an increased *G*′ at all the frequency scale, demonstrating a more viscoelastic solid-like behavior. Moreover, the sample with a 5 : 1 gelatin/PVA ratio showed the most pronounced increase, rising from 240 Pa for GP5 to nearly 450 Pa for GPS5. In formulations with lower gelatin content, the improvement was more modest, increasing from 50 Pa for GP2 to 120 Pa for GPS2. Additionally, the obtained results in a high frequency range (10–100 Hz) indicate a more elastic, and solid-like behaviour compared to the sole gelatin 5 wt% reference. As deformation increases (*i.e.* at higher frequencies) the polymer–nanoparticle interaction become more significant in governing the viscoelastic response, leading to progressively larger enhancements in *G*′. This behaviour implies that the nanocomposite structure retains its integrity even under rapid loading, rather than softening or relaxing.

The enhancement in *G*′ values for GPS samples indicates that the incorporation of SiO_2_-APTES results in significant reinforcement of the polymer matrix, which could be largely due to hydrogen bonding and physical interactions arising during the mixing phase of SiO_2_-APTES and polymers functional groups.^9,10^ Moreover, the higher *G*′ values of GP5 and GPS5 samples compared to the other samples suggests that increased gelatin content provides more functional groups for nanoparticle interaction, enabling a denser and more extensive network of polymer–nanoparticle bonds that further reinforce the material.

**Scheme 2:**
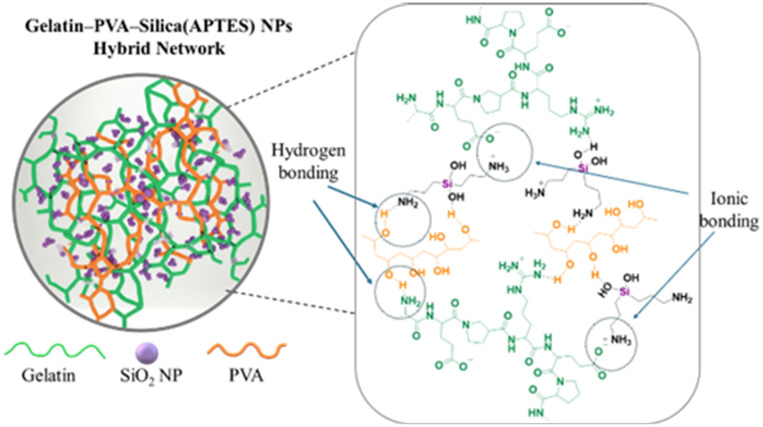
Conceptual schematic illustration of the interactions within the gelatin/PVA/SiO_2_-APTES system, showing the proposed hydrogen and ionic bonding establish between APTES residue amino groups and polymer/protein side groups form a dense hybrid network. The schematic is intended for illustrative purposes and does not represent direct molecular-level evidence.

The improved mechanical properties of the gelatin/PVA system on the other hand, have been attributed to the formation of additional hydrogen bonds and better compatibility between the two polymers. The reversible nature of these hydrogen bonds, together with the elasticity and mobility of the polymer chains, allows the material to dissipate mechanical energy efficiently through molecular motion.^27^ We hypothesize that these interactions form an interconnected network structure in which polymer chains are bound around the SiO_2_-APTES. As multiple nanoparticles simultaneously interact with several polymer chains, a continuous reinforcement network is established throughout the matrix. In other words, when SiO_2_-APTES nanoparticles are added to the gelatin/PVA mixture, several interactions occur that strengthen the adhesive network, as illustrated schematically in the gelatin–PVA–SiO_2_-APTES NPs hybrid network diagram Scheme 2. As highlighted in the schematic, extensive hydrogen bonding forms between the hydroxyl groups in PVA (orange), the functional groups in gelatin (green), and the silanol or amine groups on the SiO_2_-APTES surface, enhancing network cohesion. Similarly, gelatin/SiO_2_ interactions have been demonstrated in composite gels.^28^ Furthermore, the schematic identifies ionic bonding (electrostatic interactions) occurring between protonated amine groups (–NH_3_^+^) on the APTES and negatively charged carboxylate (–COO^−^) groups in the gelatin, further reinforcing the network.^26^ These combined hydrogen, and ionic interactions result in a denser and more interconnected network, restricting polymer chain mobility and consequently increasing the storage modulus, consistent with the reinforcing effects reported in related SiO_2_–polymer composites.^28,29^ As a result, SiO_2_-APTES addition significantly enhances the viscoelastic properties of the gelatin/PVA matrix, making it better suited for applications demanding strong adhesion.

Fig. 3(b) shows the difference in storage modulus (Δ*G*) as a function of time, demonstrating the reinforcing effect of the SiO_2_-APTES on the gelatin/PVA polymer network for different gelatin : PVA ratios 2 : 1 (Δ*G*′2), 3 : 1 (Δ*G*′3), 4 : 1 (Δ*G*′4), and 5 : 1 (Δ*G*′5). It reflects how much the presence of SiO_2_-APTES increases the storage modulus of each composition, and how that reinforcement effect behaves dynamically over the first 60 seconds. At the initial time point, the Δ*G*′ values are at their highest, particularly for the 5 : 1 gelatin : PVA ratio (Δ*G*′5), which exceeds 200 Pa. This indicates a strong initial reinforcing effect of SiO_2_-APTES, especially in gelatin-rich formulations. Over time, all four samples exhibit a gradual decrease in Δ*G*′, suggesting that part of the stiffness enhancement provided by the SiO_2_ NPs relaxes under constant oscillatory conditions. The observed decay in Δ*G*′ is likely due to the structural rearrangements within the polymer network, such as relaxation or dissociation of physical crosslinks, chain disentanglement, and partial relaxation of polymer–filler interactions, all of which reduce the effective network connectivity and lower the elastic response.^30,31^ Among the tested compositions, Δ*G*′5 maintains the highest Δ*G*′ throughout the duration of the test, implying that a higher gelatin content offers more available functional groups for effective interaction with the SiO_2_ surface. Conversely, Δ*G*′4 exhibited anomalous behaviour, starting with a negative Δ*G*′, implying that at this specific polymer ratio, the SiO_2_-APTES initially destabilize or disrupt the polymer network, leading to a temporary reduction in storage modulus before settling near a minimal difference. Triplicate measurements confirmed the reproducibility of this effect (see Fig. S5 in the SI).

Δ*G*′2 and Δ*G*′3 displayed a moderate level of reinforcement, while Δ*G*′4 shows the least enhancement, potentially due to a less optimal gelatin/PVA ratio interacting with SiO_2_-APTES. After approximately 30 to 40 seconds, all samples begin to level off, reaching a relatively stable modulus difference. This behavior may result from the SiO_2_-filled network's ability to resist additional softening and preserve its structural integrity under prolonged mechanical stress.

The shear flow analysis in Fig. 3(c and d) shows that all hydrogels both GP and GPS formulations exhibit shear-thinning (pseudoplastic) behaviour, meaning that the viscosity (*η*) decreases as shear rate (*γ̇*) increases.^32^ Results showed that increasing gelatin concentration (from GP2 to GP5) leads to higher viscosity, with GPS5 showing the greatest overall resistance. The dependence of both viscous and elastic properties (Δ*G*′, shown in Fig. 3(a and b) on the gelatin : PVA ratio confirms the development of a robust, concentration-responsive nanocomposite network. Therefore, the incorporation of SiO_2_ NPs resulted in the enhanced molecular structure of the wood adhesive and improved bonding strength.^33^ Additionally, all samples exhibited clear shear-thinning behavior, a key characteristic of effective adhesives. This property improves adhesion to the wood surface, while the observed trend could be attributed to the combined effect of higher polymer concentration and the physical reinforcement provided by SiO_2_-APTES nanoparticles, which increase chain entanglement and hinder molecular mobility. Notably, GPS5 showed the greatest elastic reinforcement and highest viscosity, making it the optimal formulation for achieving maximum bonding performance in structural wood joints. The viscous component of the viscoelastic response was further examined through the loss modulus (*G*″) and damping factor (tan *δ*), and the corresponding results are presented in the SI (Fig. S6(a and b)).

### Adhesion strength of gelatin/PVA adhesives on wood

**Fig. 4:**
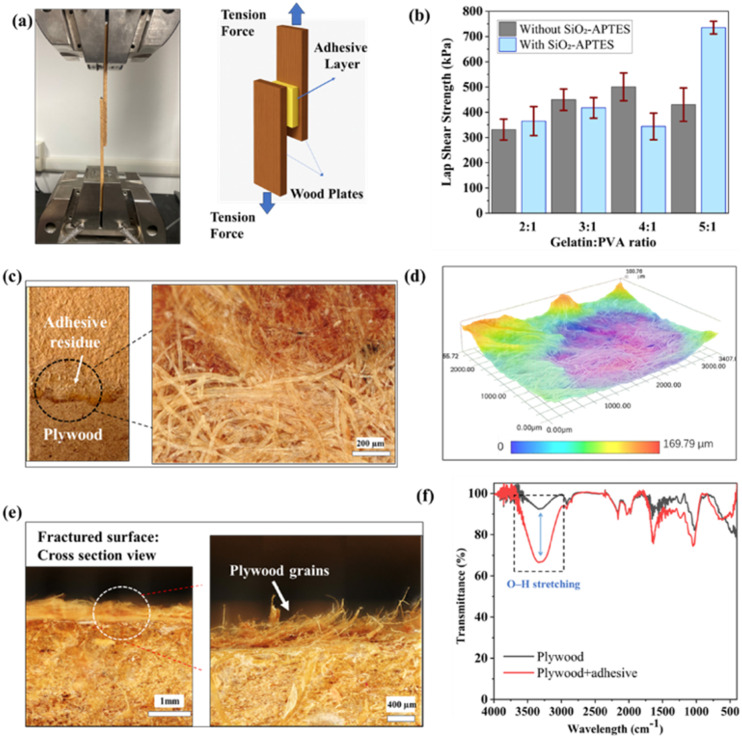
Effect of SiO_2_-APTES NPs on gelatin/PVA adhesive performance: (a) lap shear test setup, (b) shear strength comparison (data are presented as mean ± SD, *n* = 3). (c) Optical microscopy images of the plywood surface after failure, showing adhesive residue and wood fiber morphology. (d) Corresponding 3D surface topography map of (c) showing the distribution and roughness of the interface. (e) Cross-sectional views of the plywood surface after failure, showing plywood grains and the bonded interface (f) FTIR spectra of plywood and plywood treated with adhesive.

The hybrid gelatin/PVA/SiO_2_-APTES systems are tested as adhesives on plywoods. We have tested the lap-shear strength to find out the optimized system for wood adhesion. Fig. 4(a) illustrates the schematic setup used for the lap shear test to evaluate the adhesion strength of wood adhesives. Two overlapping wood plates are bonded by prepared bio adhesives and opposing forces are applied along the length of the plates to generate shear stress within the joint. The maximum load sustained before failure represents the adhesive's shear strength, providing a measure of its bonding performance. The lap shear strength values for each sample are presented in Fig. 4(b). All SiO_2_-reinforced materials exhibit an increase in shear strength, ranging from 364 ± 57 to 735 ± 25 kPa. Specifically, the incorporation of SiO_2_-APTES in GP5 results in a shear strength of 735 ± 25 kPa, corresponding to an approximately 71% increase over its unfilled counterpart (429 ± 66 kPa). This could be due to the enhanced compatibility between the polymer matrix and SiO_2_-APTES by increasing the gelatin content resulted in a significant increase in shear strength, indicating more efficient stress transfer across the bonded interface. However, GP2, GP3, and GP4 do not show a significant enhancement in shear strength. The improvement was not even observed for GP3 and GP4, as the incorporation of SiO_2_ NPs led to a slight decrease in strength. It might be attributed to the weaker interactions with the polymer matrix at intermediate gelatin/PVA compositions. In these formulations, SiO_2_-APTES particles may disrupt the polymer network rather than reinforce it. The SiO_2_-APTES reinforcement successfully transforms the gelatin/PVA system from a low-strength hydrogel to a robust adhesive, although its final strength is distinct from the upper class of highly hardened biopolymer and synthetic wood adhesives. While higher gelatin content (5 : 1) improved adhesion by enhancing particle dispersion and interfacial cohesion. Overall, the adhesiveness improved with increasing gelatin content, likely due to the higher concentration of charged amino acid residue, such as arginine, aspartic acid, and glutamic acid, which enhance electrostatic cohesion between macromolecules. These findings demonstrate that nanoparticle-mediated reinforcement effectively enhances interfacial adhesion to plywood, placing the formulation in a technologically relevant intermediate range between low-strength gelatin/PVA hydrogels and highly crosslinked industrial gelatin adhesives.

From a performance perspective, the obtained lap-shear strength values in this study (735 ± 25) kPa are lower than those reported for conventional wood adhesives such as urea-formaldehyde (UF) and phenol-formaldehyde (PF), which typically exhibit strengths in the range of 5–10 MPa under dry conditions.^34,35^ However, this difference is primarily attributed to the distinct curing conditions, as industrial UF and PF systems require high-temperature hot-pressing to achieve optimal crosslinking density, whereas the present system was evaluated as a cold-setting adhesive under ambient conditions. The measured strength is within the range reported for bio-based adhesives cured under mild or ambient conditions, which generally exhibit shear strengths below 1–1.5 MPa depending on formulation and processing conditions.^36^ Results demonstrate a promising baseline for energy-efficient, cold-setting bio-based adhesive systems.

Post-fracture surface analysis reveals cohesive failure within the plywood substrate, as evidenced by visible adhesive GPS5 residues (Fig. 4c). The corresponding surface topography in Fig. 4d further indicates a heterogeneous morphology at the microscale, suggesting localized interlocking features and confirming that failure occurs within the wood rather than at the adhesive interface. Optical microscopy of the cross-sectional view (Fig. 4e) further highlights the rough, fibrous structure of the fractured surfaces, with pronounced fiber pull-out, indicating effective adhesive penetration and strong interaction with the wood substrate. Complementary ATR-FTIR spectra of plywood and plywood-adhesive systems (Fig. 4f) show a broad absorption band in the 3200–3600 cm^−1^ range, corresponding to O–H stretching vibrations. After adhesive incorporation, this band becomes noticeably broader and more intense, indicating enhanced hydrogen bonding interactions. This suggests the formation of intermolecular hydrogen bonds between hydroxyl groups of the wood substrate and functional groups within the adhesive.

**Fig. 5:**
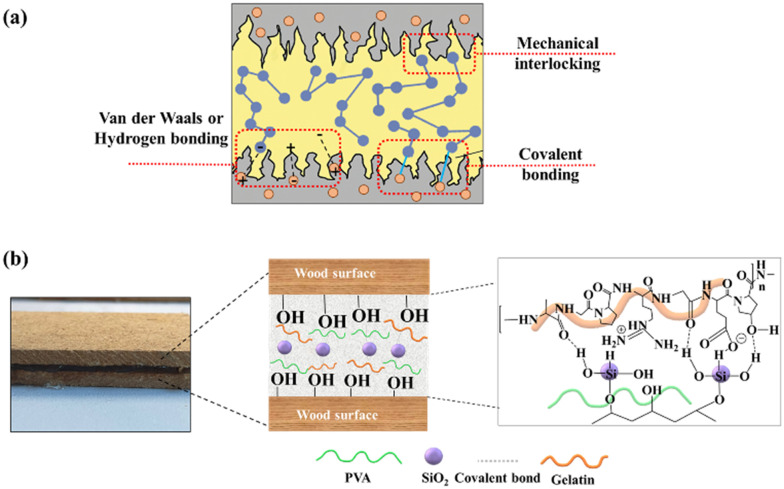
(a) Schematic representation of adhesive bonding strengths. (b) Proposed schematic of molecular interactions between the wood surface and the SiO_2_-filled gelatin/PVA adhesive.

Based on the above experimental evidence, a plausible adhesion mechanism is proposed and illustrated in Fig. 5. Fig. 5(a) shows the adhesive bonding strengths including mechanical bonding, which arises from physical interlocking with the surface roughness, whereas chemical bonding involves primary molecular interactions between the adhesive and the substrate.^37^ Based on these mechanisms, the proposed interactions between the formulated adhesive (gelatin/PVA/SiO_2_-APTES) and the wood substrates are depicted in Fig. 5(b). The wood substrate is rich in hydroxylated polysaccharides (cellulose and hemicellulose) and phenolic lignin, all of which provide abundant surface hydroxyl (–OH) groups.^38^ Hydrogen bonding between the SiO_2_ surface and the wood substrate, combined with possible mechanical interlocking enabled by the presence of SiO_2_-APTES NPs, can create a more integrated and chemically bonded interface between the polymer–nanoparticle adhesive and the wood substrate. This synergistic effect explains the observed increase in shear strength upon the addition of SiO_2_-APTES. Furthermore, interactions within the PVA/gelatin adhesive matrix contribute to enhanced adhesive performance. The results suggest that the beneficial effect of SiO_2_ NPs strongly depends on the matrix composition and the balance between polymer–polymer and polymer–filler interactions. In formulations with sufficiently high gelatin content, the nanoparticles act as additional physical crosslinkers, reinforcing interfacial adhesion and facilitating more efficient load transfer. Conversely, in mixtures with lower gelatin content, the incorporation of SiO_2_ NPs may disrupt the native hydrogen-bonding network, thereby weakening the cohesive strength of the matrix and reducing overall lap shear performance. This dual behavior highlights the importance of simultaneously optimizing filler content and matrix composition rather than considering them independently.

## Conclusions

The incorporation of SiO_2_-APTES into gelatin/PVA hydrogels markedly improved their rheological and adhesive properties. The increase in storage modulus (*G*′) across all formulations indicates the development of a more elastic and highly crosslinked polymer network, arising from covalent Si–O–C bonds, hydrogen bonding, and electrostatic interactions between the SiO_2_-APTES NPs and the functional groups of the polymers. These interactions restrict polymer chain mobility, enhance network densification, and provide improved viscoelastic stability over a broad frequency range. Shear flow analysis revealed pronounced shear-thinning behavior, while the observed increase in viscosity reflects stronger internal cohesion and improved interfacial compatibility within the composite system. Lap shear testing further demonstrated that nanoparticle incorporation significantly enhanced dry adhesion strength on wood substrates, particularly in gelatin-rich formulations (5 : 1 gelatin : PVA). In this composition, adhesion strength increased by 122% compared to the 5 wt% gelatin-based adhesive and by 71% relative to the unfilled 5 : 1 gelatin/PVA system. This enhancement is attributed to the combined effects of mechanical interlocking and chemical bonding at the adhesive–wood interface. Overall, SiO_2_-APTES-reinforced gelatin/PVA hydrogels represent a robust, tunable and sustainable bio-based adhesive platform suitable for high-performance bonding applications while minimizing the need for additional toxic chemical reagents.

Finally, it should be noted that the present adhesive system is expected to exhibit sensitivity to moisture due to the hydrophilic nature of gelatin and PVA, which may affect long-term performance under humid or aqueous conditions. It is well established that water uptake in hydrophilic polymer networks can lead to plasticization, swelling, and a subsequent reduction in mechanical strength over time.^39^ Moreover, while accelerated aging and humidity cycling tests were not performed in this study, this represents a limitation that should be addressed in future work to fully evaluate the long-term durability and water resistance of the developed adhesive systems under realistic service conditions.

## Author contributions

Sogand Abbaspoor-Zanjani: investigation, formal analysis, writing – original draft. Carlo Di Bernardo: methodology, validation. Jenny Flores Garcia: data curation. Camilla Noè: review and editing. Mengjiao Wang: review and editing. Massimo Messori: resources, supervision, funding acquisition, review and editing. Teresa Gatti: conceptualization, resources, supervision, funding acquisition, review and editing.

## Conflicts of interest

There are no conflicts to declare.

## Supplementary Material

LF-003-D6LF00152A-s001

## Data Availability

Additional datasets supporting this work have been included as part of the supplementary information (SI). The datasets generated and analyzed during this work can be made available from the authors on request. Supplementary information is available. See DOI: https://doi.org/10.1039/d6lf00152a.
