# Supplementary material for: Functionalized nano SiO2 reinforced gelatin–PVA hydrogels for sustainable wood adhesion
Source: RSC Appl Interfaces. 2026 May 28;3(4):1198–207. doi: 10.1039/d6lf00152a (PMC13245114; doi:10.1039/d6lf00152a)
Supplement: LF-003-D6LF00152A-s001 [file LF-003-D6LF00152A-s001.pdf]

Supplementary Information (SI) for RSC Applied Interfaces.
This journal is © The Royal Society of Chemistry 2026

Supplementary Information for:

Functionalized Nano SiO_2 Reinforced Gelatin–PVA Hydrogels for Sustainable Wood Adhesion

Sogand Abbaspoor-Zanjani,[a*] Carlo Di Bernardo,[a] Jenny Flores Garcia,[a] Mengjiao Wang,[a] Massimo Messori[a], Camilla Noè[a*] and Teresa Gatti[a*]

[a] *Department of Applied Science and Technology, Politecnico di Torino, Corso Duca degli Abruzzi 24, 10129 Torino, Italy*

SI1. Roughness measurement

Table S1. Confocal roughness parameters (Sa, Sq, Sz) of the plywood substrate measured at three locations (ISO 25178; plane leveling; λs = 5 µm, λc = 50 µm).

Measurement	Sa (µm)	Sq (µm)	Sz (µm)
1	2.25	3.38	137.5
2	2.11	3.46	144.6
3	1.71	3.39	140.8

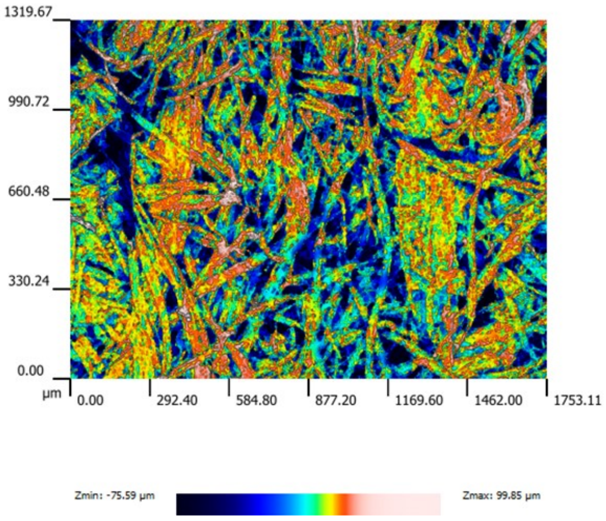

Fig S1. Representative confocal 2D surface map of the plywood substrate (same processing conditions).

SI2. FTIR analysis of nanoparticles

The full FTIR spectra of pristine SiO_2 and SiO_2–APTES NPs are shown in Fig. S2. Both samples display the characteristic Si–O–Si vibrations of the silica framework around 1000–1250 cm^{-1} and ~800 cm^{-1}, as well as a broad O–H stretching band at 3200–3700 cm^{-1}. In the SiO_2–APTES spectrum, additional bands

in the 2850–2950 cm^{-1} and 1550–1650 cm^{-1} regions corresponding to C–H and N–H vibrations confirm the presence of APTES on the silica surface.

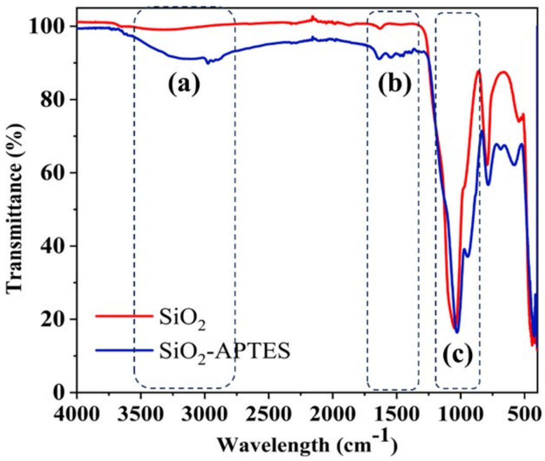

Fig. S2 Full FTIR spectra of SiO_2 and SiO_2-APTES NPs

SI3. Thermogravimetric Behavior of APTES functionalized SiO_2

As shown in Fig. S2, both samples exhibited an initial weight loss below 150 °C, attributed to the desorption of physically adsorbed moisture. However, the SiO_2-APTES NPs displayed a higher moisture loss in this region (approximately 10–12%) compared to bare SiO_2 (about 2–3%), indicating enhanced water adsorption of APTES due to surface functionalization. Beyond 150 °C, bare SiO_2 displayed a gradual and minor mass decrease, consistent with the high thermal stability of silica. In contrast, SiO_2-APTES exhibited a continuous and more pronounced weight loss up to 800 °C, which is consistent with the presence of an APTES layer, which enhances water absorption.[1]

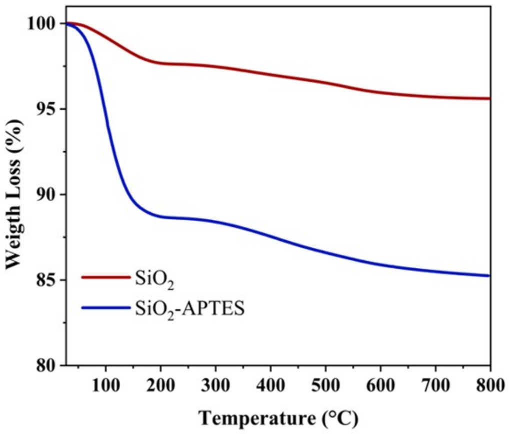

Fig. S3 TGA of bare SiO_2 and SiO_2-APTES NPs

SI4. Nitrogen adsorption–desorption (BET) analysis

Nitrogen adsorption–desorption measurements were performed at 77 K to evaluate the textural properties of SiO_2 and SiO_2–APTES. The specific surface area was calculated using the BET method, and pore characteristics were obtained from BJH analysis. The bare SiO_2 NPs exhibited a BET surface area of 5.01 m^2 g^{-1}, which decreased to 3.61 m^2 g^{-1} after APTES functionalization, indicating partial coverage of the silica surface by aminopropyl groups grafted onto surface silanol sites.[2] The pore volume also decreased after modification, confirming successful surface grafting. The nitrogen adsorption–desorption isotherms and the corresponding textural parameters are presented in Fig S3.

Table S2. BET surface area and pore characteristics of SiO_2 and SiO_2–APTES NPs derived from nitrogen adsorption–desorption measurements.

Sample	SBET ($m^2\ g^{-1}$)	Vtotal($cm^3\ g^{-1}$)	Average pore width (nm)
SiO_2	5.01	0.00716	30.78
SiO_2–APTES	3.61	0.00299	11.46

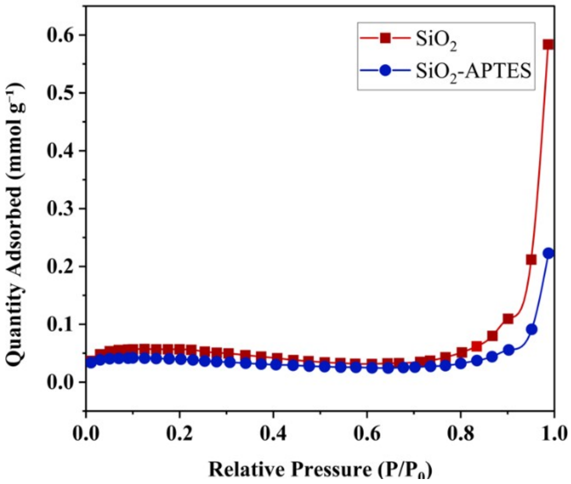

Fig. S4. Nitrogen adsorption–desorption isotherms of SiO_2 and SiO_2–APTES NPs measured at 77 K.

SI5. Rheological analysis

Fig. S5 displays the ΔG' curves for three independent replicates of GP4 and GPS4 (4:1) formulations, confirming the consistency of the destabilization effect at the 4:1 ratio.

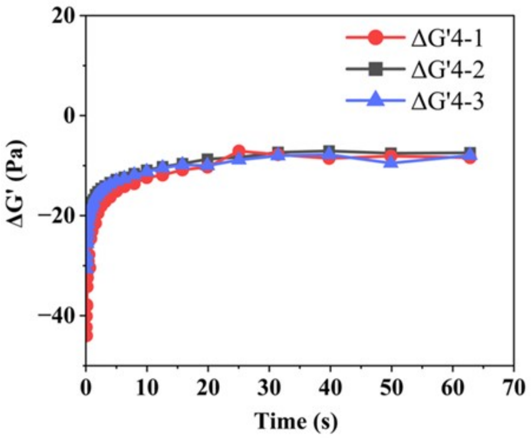

Fig. S5 Reproducibility study for rheological reinforcement of gelatin/PVA adhesive formulations at at the 4:1 ratio.

The loss modulus (G″) as a function of frequency for the gelatin/PVA formulations with and without SiO_2-APTES NPs is presented in Fig. S4a. In general, G″ increases with increasing frequency for all samples, reflecting the typical viscoelastic response of polymer-based adhesive systems. The incorporation of SiO_2=APTES NPs slightly modify the viscous response of the system and leads to comparable or moderately higher G″ values in several compositions compared to the corresponding GP formulations. This behavior suggests that the presence of SiO_2-

APTES NPs contribute to additional molecular interactions within the polymer network, affecting the energy dissipation characteristics of the nanocomposite system, as reported for nanoparticle-reinforced gelatin/PVA composites.[3]

The damping factor (tan δ = G″/G′) as a function of frequency is shown in Fig. S4b. All samples exhibit tan δ values well below unity across the investigated frequency range, indicating that the elastic component dominates over the viscous response. Moreover, the GPS formulations generally display slightly lower tan δ values compared to the corresponding GP samples, suggesting that the incorporation of SiO_2-APTES enhances the elastic character of the network. This reduction in tan δ is consistent with stronger polymer–nanoparticle interactions and a more interconnected viscoelastic structure within the composite adhesive system.

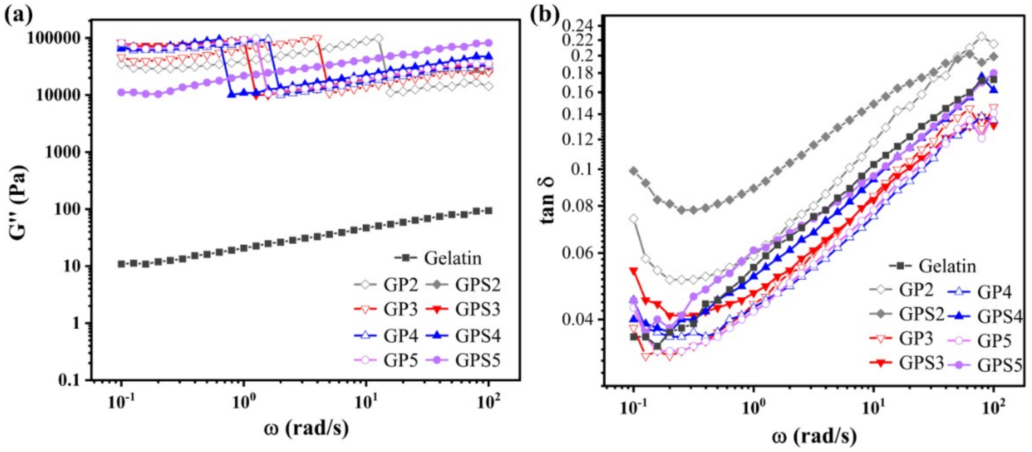

Fig. S6 (a) Frequency dependence of the loss modulus (G″) for gelatin/PVA adhesive formulations with and without SiO_2–APTES NPs. (b) Damping factor (tan δ = G″/G′) as a function of angular frequency for the corresponding systems, illustrating the relative contributions of viscous and elastic responses

SI6. Fractured Surface

The specimens were further separated manually after testing to expose the fracture surfaces. The images show extensive fiber pull-out and the absence of a clean interfacial separation for GPS5, indicating cohesive failure within the wood substrate. This suggests that the adhesive-wood interface is stronger than the cohesive strength of the wood, reflecting effective interfacial bonding, while for GP5 the adhesive was separated, showing weaker adhesive-wood interface.

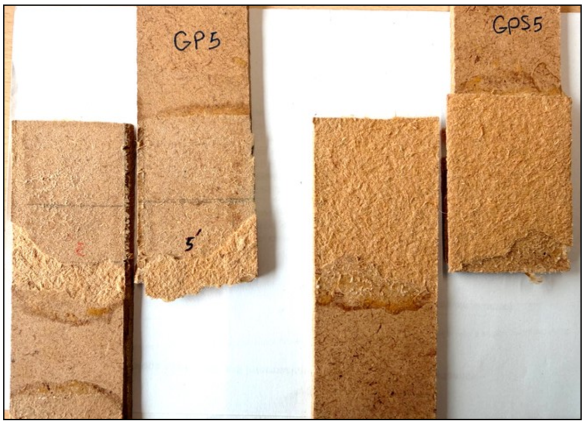

Fig S7. Fracture surfaces of plywood specimens after lap-shear testing for GP5 and GPS5 adhesives

SI7. Microscopic analysis of adhesives

Optical microscopy of GPS5 adhesive (Fig. S8) shows a largely homogeneous microstructure, with minor localized heterogeneities observed.

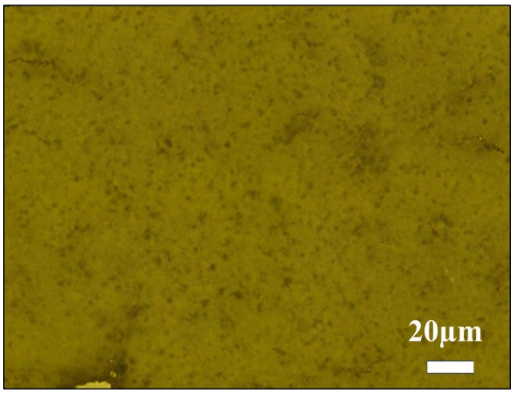

Fig S8. Optical microscopy image of GPS5 adhesive-1000x

References

1 M. Sándor, C. L. Nistor, G. Szalontai, R. Stoica, C. A. Nicolae, E. Alexandrescu, J. Fazakas, F. Oancea and D. Donescu, Aminopropyl-Silica Hybrid Particles as Supports for Humic Acids Immobilization, *Materials (Basel)*, DOI:10.3390/MA9010034.

2 L. T. Zhuravlev, The surface chemistry of amorphous silica. Zhuravlev model, *Colloids Surf. A Physicochem. Eng. Asp.*, 2000, **173**, 1–38.

3 T. M. Temel-Soylu, C. Keçeciler-Emir, T. Rababah, C. Özel, S. Yücel, Y. Basaran-Elalmis, D. Altan, Ö. Kirgiz, İ. E. Seçinti, U. Kaya and M. E. Altuğ, Green Electrospun Poly(vinyl alcohol)/Gelatin-Based Nanofibrous Membrane by Incorporating 45S5 Bioglass Nanoparticles and Urea for Wound Dressing Applications: Characterization and In Vitro and In Vivo Evaluations, *ACS Omega*, 2024, **9**, 21187–21203.
